# Underwater endoscopic submucosal dissection using a novel extended projection cap for colorectal tumors

**DOI:** 10.1055/a-2740-3788

**Published:** 2025-12-11

**Authors:** Tomoya Ueda, Noriya Uedo, Koji Higashino, Kenneth Binmoeller

**Affiliations:** 1 53312 Department of Gastrointestinal Oncology, Osaka International Cancer Institute, Osaka, Japan; 2 38049 Department of Gastroenterology and Hepatology, Kyoto University Graduate School of Medicine, Kyoto, Japan; 3 Interventional Endoscopy Services, California Pacific Medical Center, San Francisco, California, United States

Underwater endoscopic submucosal dissection (UESD) provides a clear, magnified view without lens clouding. Furthermore, the water pressure delivered by a water-jet system facilitates submucosal dissection by widening the submucosal layer, even in narrow spaces with fibrosis 1 2 3 4 . However, during UESD, the visual field can be impaired by bubbles which are generated by electrosurgical discharge in normal saline 5 . This problem is particularly evident when using a tapered hood, as the bubbles tend to be trapped inside the hood. Herein, we present a case of colonic UESD using a novel extended projection cap (Visor Cap; VC41124, Micro-tech, Nanjing, China), which features a semi-circumferential tapered visor with a small hole at its base ( Fig. 1 ).

**Fig. 1:**
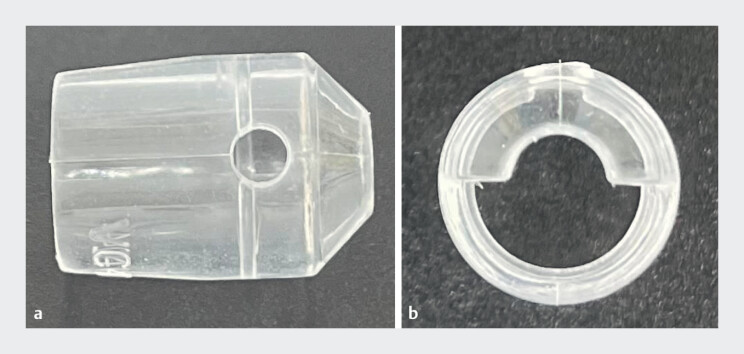
**a, b** The visor cap: a novel extended projection cap featuring a semi-circumferential tapered visor with a hole at its base, designed to facilitate submucosal dissection by providing effective counter-traction and maintaining the bubble-free view under the underwater conditions.

A 59-year-old man was referred for management of a 25-mm laterally spreading tumor located in the ascending colon ( Fig. 2 ). UESD was performed using a colonoscope (PCF-H290ZI; Olympus, Tokyo, Japan) equipped with the visor cap ( Video 1 ). After circumferential mucosal incision was made using a FlushKnife BT-S (Fujifilm, Tokyo, Japan), the initial mucosal flap was easily created by applying the visor just beneath the mucosa ( Fig. 3 **a** ). Even when pulsatile bleeding occurred, reliable hemostasis was obtained under gas conditions using hemostatic forceps through its lower semi-circumferential opening ( Fig. 3 **b** ). During submucosal dissection, the visor provided effective counter-traction by lifting the overlying mucosa ( Fig. 3 **c** ). Furthermore, the small hole at the base of the visor, along with the semi-circumferential opening, allowed bubbles to escape easily with the aid of a water jet. *En bloc* resection was successfully achieved without adverse events ( Fig. 4 ). Pathological examination of the resected specimen revealed intramucosal, well-differentiated tubular adenocarcinoma with negative margins.

**Fig. 2:**
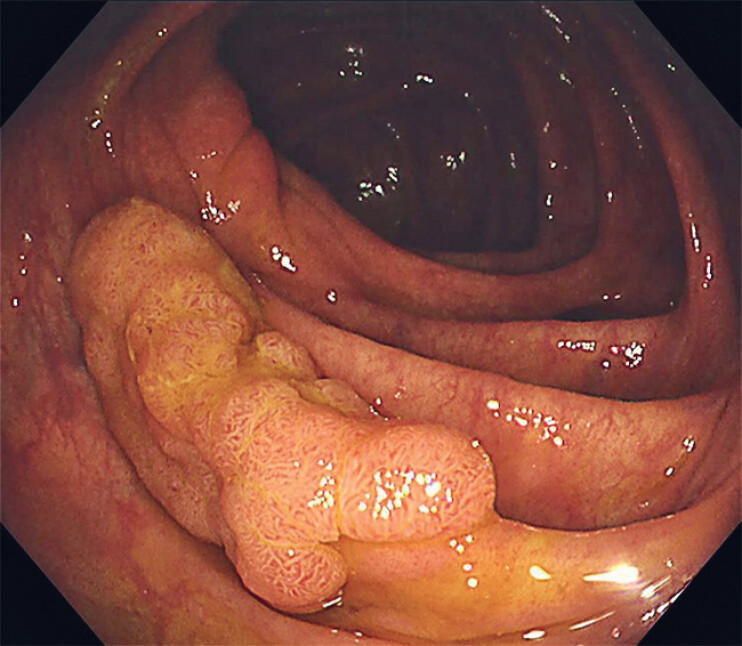
Endoscopic submucosal dissection was performed for a 25-mm laterally spreading tumor located in the ascending colon.

Underwater endoscopic submucosal dissection using a novel “visor cap”.Video 1

**Fig. 3:**
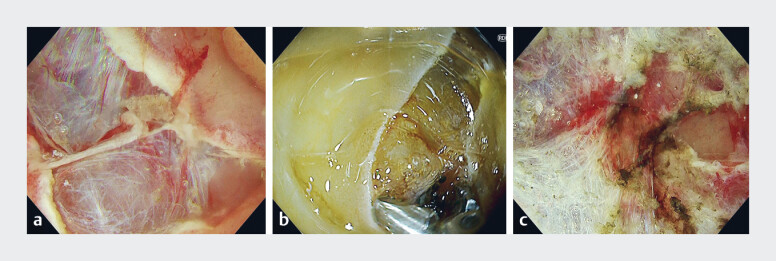
Endoscopic view during endoscopic submucosal dissection. **a** The initial mucosal flap was easily created by applying the visor just beneath the mucosa. **b** Reliable hemostasis was obtained under gas conditions using hemostatic forceps through its lower semi-circumferential opening. **c** The visor provided effective counter-traction by lifting the overlying mucosa.

**Fig. 4:**
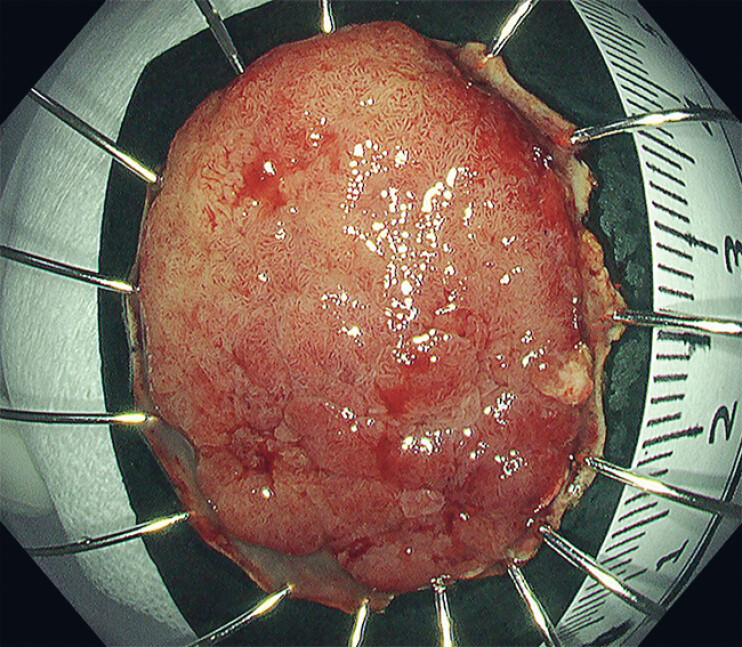
*En bloc* resection was successfully achieved without adverse events.

In conclusion, this novel visor cap provides effective counter-traction and helps to maintain a clear, bubble-free view under underwater conditions without compromising the maneuverability of ESD devices.

Endoscopy_UCTN_Code_TTT_1AQ_2AD_3AZ

## Notes

Endoscopy E-Videos https://eref.thieme.de/e-videos
*E-Videos* is an open access online section of the journal *Endoscopy* , reporting on interesting cases and new techniques in gastroenterological endoscopy. All papers include a high-quality video and are published with a Creative Commons CC-BY license. Endoscopy E-Videos qualify for HINARI discounts and waivers and eligibility is automatically checked during the submission process. We grant 100% waivers to articles whose corresponding authors are based in Group A countries and 50% waivers to those who are based in Group B countries as classified by Research4Life (see: https://www.research4life.org/access/eligibility/ ). This section has its own submission website at https://mc.manuscriptcentral.com/e-videos .
